# 

**Published:** 1887-07

**Authors:** 


### EDITORIALS AND MISCELLANEOUS.

### TO OUR SUBSCRIBERS.

Many of our subscribers are in arrears for the RECORD. Just at this writing we have not time to send statements of account. Let every one who knows he has not paid make us a remittance for at least one year, and in acknowledging receipt of same we will send statement of any balance existing. Money is the oil which lubricates the machinery and keeps our business going. Don't wait for a formal dun, but send something, as we need a little just now, and as soon as possible bills will be sent to all.

### RECEIPTED.

1887—J. S. Horseley, A. D. Coleman, to May; W. R. Greenlee, to June; S. S. Barnes, L. W. Pitchford, G. B. Lawrence, to June, 1888; E. A. Perkins, to June, 1888; B. L, Clifton, to June, 1888.

1887—R. D. Smoot, Wm. Wordlaw; James Rivers, T. R. Robarts, J. L. Simpson, T. T. Martin, G. W. Keller.

